# Sun Exposure and Melanoma, Certainties and Weaknesses of the Present Knowledge

**DOI:** 10.3389/fmed.2018.00235

**Published:** 2018-08-30

**Authors:** Mariachiara Arisi, Cristina Zane, Simone Caravello, Chiara Rovati, Arianna Zanca, Marina Venturini, Piergiacomo Calzavara-Pinton

**Affiliations:** Department of Dermatology, Spedali Civili di Brescia, University of Brescia, Brescia, Italy

**Keywords:** melanoma, sun exposure, vitamin D, UVA, UVB

## Abstract

Sun exposure is the main risk factor for cutaneous malignant melanoma (CMM). However, the UV-related pathogenetic mechanisms leading to CMM are far to be fully elucidated. In this paper we will focus on what we still don't fully know about the relationship between UVR and CMM. In particular, we will discuss: the action spectrum of human CMM, how different modalities of exposure (continuous/ intermittent; erythemal/ suberythemal) relate to different CMM variants, the preferential UVR induced DNA mutations observed in different CMM variants, the role of UV-related and UV-unrelated genetic damages in the same melanoma cells. Moreover, we will debate the importance of UVA induced oxidative and anaerobic damages to DNA and other cell structures and the role of melanins, of modulation of innate and acquired immunity, of vitamin D and of chronic exposure to phototoxic drugs and other xenobiotics. A better understanding of these issues will help developing more effective preventative strategies and new therapeutic approaches.

## Introduction

It is widely accepted that ultraviolet light radiation (UVR) is the major—but not the only—risk factor for the development of cutaneous malignant melanoma (CMM) (1). It is thought that genotoxic, inflammatory, and immunosuppressive properties of UVR contribute together to initiation, progression, and metastasis of CMM. However, several important mechanistic details regarding how sunlight causes CMM remain to be fully elucidated. As a consequence, we still cannot provide fully effective preventative behavioral strategies. In the present paper, we will focus on the main weaknesses of the present understanding of UVR-CMM relationships.

## Modality of UVR exposure and CMM variants

Cutaneous malignant melanoma (CMM) is not a single tumor entity with a homogeneous profile of risk factors and prognosis. Consequently we recognize a few variants. It is completely unclear why different modalities of UVR exposure (erythemal/suberythemal doses; chronic/intermittent exposures) induce different molecular damages in the same cell population (2) and why these different molecular damages lead to different clinical CMM variants. For example, we do not know why chronic lifetime sun damage, seen in elderly people and outdoor workers, is related to the specific pattern of DNA mutations characteristic of Lentigo Maligna Melanoma (LMM) (3), while sunburns do not seem to be a significant risk factor for this CMM variant (4). In contrast, Superficial Spreading Melanoma (SSM) and Nodular Melanoma (NM), that have a different spectrum of UV related DNA mutations, usually develop on intermittently exposed healthy skin of younger subjects (3) and a history of sunburns (particularly during childhood) was found to double the risk (5).

## Waveband dependency of genotoxic damages and CMM

**Figure 1:**
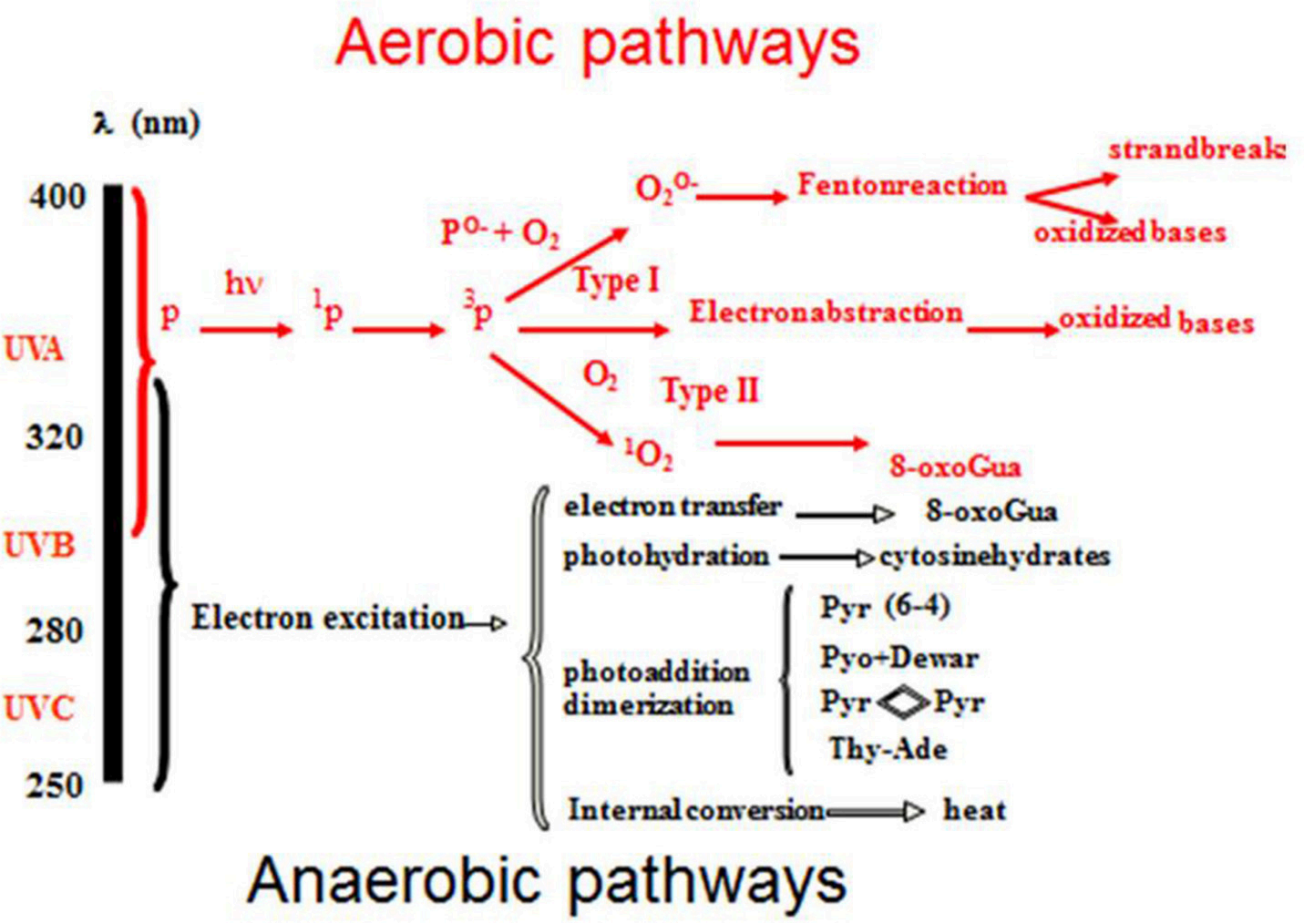
UV- induced DNA damages and their waveband dependency.

The most relevant cromophore for skin carcinogenesis is DNA. Its absorption peak is in the UVB region. The different types of UV-DNA photoproducts and their waveband- dependency are summarized in Figure 1. Cyclo-butane pyrimidine dimers (CPD) (T < >T, C < >C, C < >T, and T < >C)-and 6–4 photoproducts (6–4PP) are the most frequent. Until few years ago, it was assumed that UVB biological effects were mainly caused by oxygen-independent reactions, whereas UVA reactions were considered exclusively oxygen dependent. As a consequence, the terms “UVB effects” and “UVA effects” were used as synonyms for anaerobic (synonyms: direct, anoxic or type 1) and aerobic (synonyms: indirect, oxidative or type 2) effects, respectively. However, it was demonstrated that UVA induces DNA type 1 damages as well (6). Indeed, UVA seems able to produce oxidative DNA damage directly or after the oxidative sensitization of not yet identified endogenous photosensitizers (7). Concerning DNA damages, it is therefore clear that the sharp distinction between UVB and UVA, should be reconsidered, because C -> T transitions and CC -> TT tandem mutations (8) are no more to be considered as only UVB damages. Therefore, the term “UVB signatures” should be avoided because potentially misleading.

UVA genotoxic activity is about 1,000 times weaker than UVB's one if considered on a per photon basis (6–8). However, its importance is partially compensated by the UVA environmental irradiance that is about 20–40 times higher, depending on some factors, including time of day, season, latitude and altitude (9). In addition, UVA irradiation is even higher when UV exposure happens through a window glass or in sunbeds. Also, the application of non-broad-spectrum sunscreens, not able to filter UVA as much as UVB, can cause UVA over- exposure. In the same way, aerobic damages to DNA, leading to the formation of 8-oxo-7,8-dihydro-2′deoxyguanosine (8oxo-dG) and consequent reparation with G → T transversions and G → A transitions, cannot be considered “UVA signatures” because UVB is able to produce aerobic damages as well (6, 8).

Other mutations, induced by both UVA and UVB, are DNA-protein cross-links and single and double strand breaks, but their role in CMM development remains to be clarified (6, 8). Anaerobic UV DNA damages are repaired by the nucleotide excision repair (NER) system, while oxidative DNA damages are repaired by the base excision repair (BER) system. It is interesting to note that the mutation rate per DNA photoproduct is higher with UVA: in fact UVA damages are not followed by as many protective, anti-mutagenic and reparative responses as UVB damages are (10).

**Figure 2:**
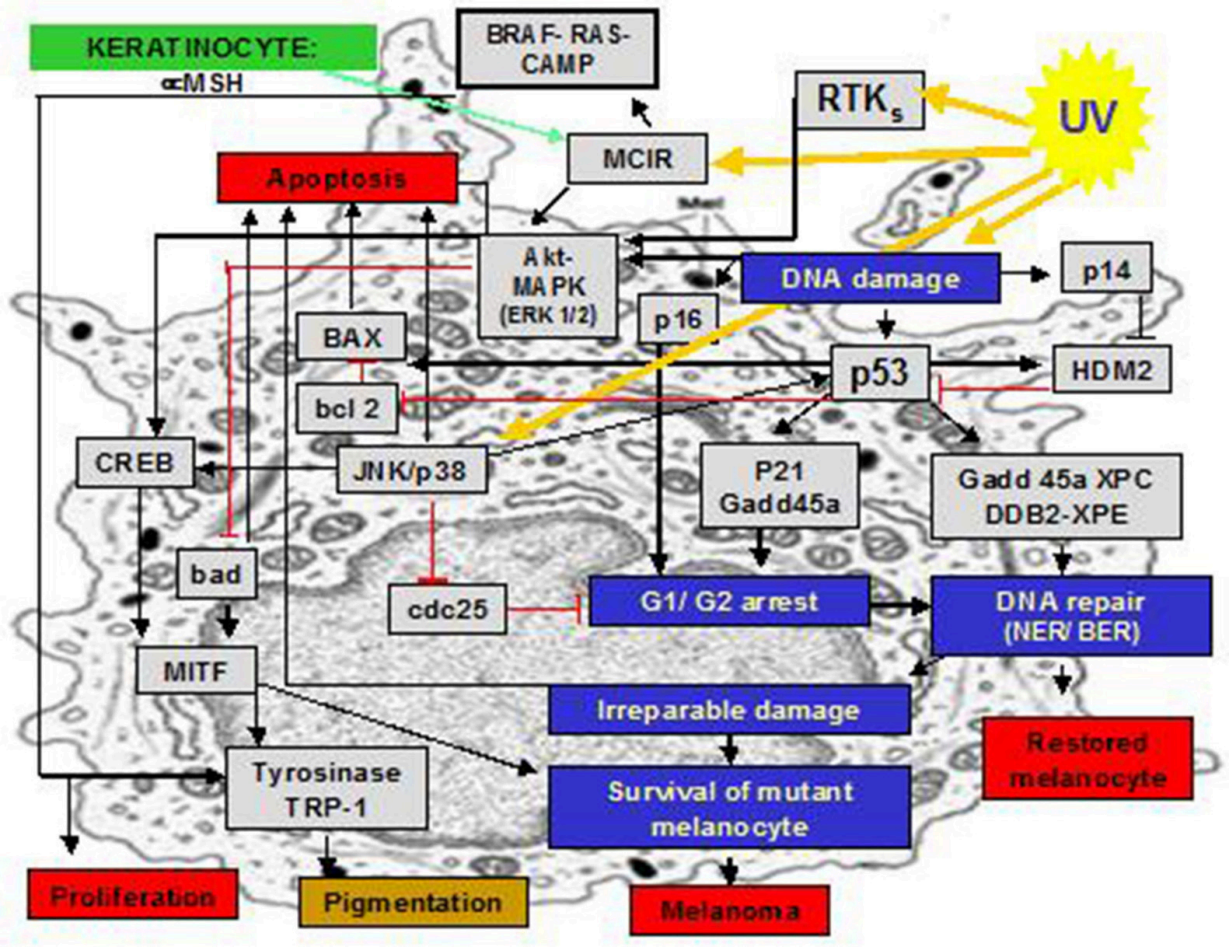
Selected inflammatory molecular pathways that are triggered by UVR in melanocytes.

Anaerobic and aerobic photoproducts of DNA, together with other biomolecules, induce a cascade of pro-inflammatory signals and suppress pro apoptotic pathways (Figure 2). However, the respective relevance and interplay of UVA- and UVB- activities are still largely unknown. Finally, we emphasize how important it is to extrapolate in a very careful and critical manner the results of experimental studies about photo-genotoxicity made with cell cultures: during “*in-vivo*” UV exposure, while UVA has a deeper penetration, only a small fraction of the incident UVB radiation reaches the level of the dermal-epidermal junction of human skin, where melanocytes are located. We should also be careful with findings of studies on animals, because penetration into human skin may be different.

## UVR and gene mutations

Gene mutations found in CMM cells are more frequent at selected loci. Aiming to understand their possible diagnostic and prognostic meaning, they have been divided in two main groups: mutations providing no selective advantage to the tumor growth (that occur stochastically during cancer development), and genomic alterations that have a role in cancer development or in the determination of cancer phenotype. In order to assess a correlation between sun exposure and mutations at hot-spots within promoters, the detection of canonical UV signatures (C to T and CC to TT mutations) is mandatory.

UVR-induced mutations are frequently found in the CDKN2A gene in all CMM variants. In humans, this gene encodes for the tumor suppressor proteins p16 and p14ARF (11).

N-RAS is the most frequently affected RAS family member in CMM. Both anaerobic and oxidative, as well as non-UV related, damages have been detected on this gene (12). These mutations can lead to the production of a permanently activated RAS protein, causing the consequent activation of phosphatidylinositol 3′ kinase and mitogen-activated protein kinase (MAPK) pathways. The constant activation of these two pathways leads to unintended and overactive signaling for cell growth, differentiation and survival even in the absence of incoming signals (13). If the frequency of NRAS mutations in LMM, in comparison to SSM and NM, is higher (14, 15) or not (16, 17) is still debated.

The BRAF gene encodes for a serine/threonine kinase that plays a key role in the MAPK signaling pathway. BRAF mutation is more often associated with SSM and NM and it is particularly common in younger patients (18–22). KIT mutations are often found in acral lentiginous melanoma (ALM) and mucosal melanoma (MuM), less frequently in LMM, and rarely in SSM and NM (23). The gene encoding for the pro-apoptotic p53 is another frequent target of UVB damage. Mutated p53 is often observed in melanoma metastases (24), while it is less frequent in primary CMM. This clearly indicates a role for UVR in CMM progression (25, 26). The Nucleotide Excision Repair (NER), and in particular the global genome repair (GGR) damage recognition sub-group, may also be damaged by UVR in melanoma cells, leading to a defective DNA repair response (27, 28).

**Figure 3:**
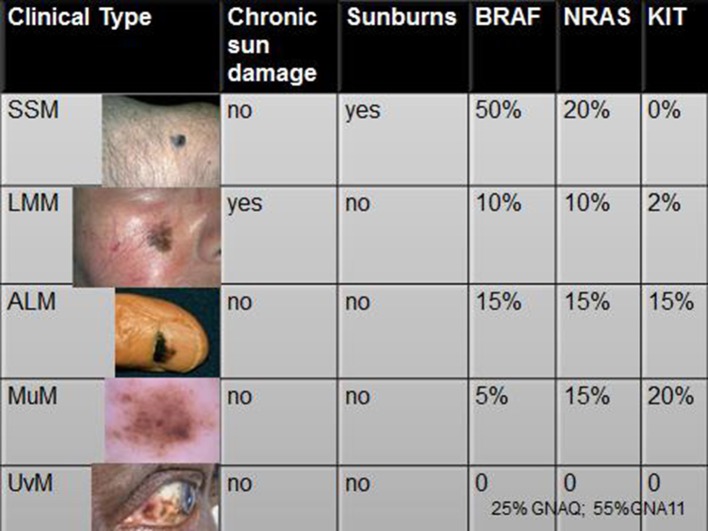
Associations of the clinical variants of CMM with modalities of exposures and driver mutations. Legend: SSM, superficial spreading melanoma; LMM, Lentigo Maligna Melanoma; ALM, acral lentiginous melanoma; MuM, mucosal melanoma; UvM, uveal melanoma (references in the text).

Therefore, we can conclude that clinical variants of CMM are differently associated to different driver mutations (Figure 3). However, the biological mechanism for which selected DNA mutations drive the mutated melanocyte to a specific clinical variant of CMM is unknown. Furthermore, a significant correlation between clinical outcome and genomic damages is yet to be found (29).

## UV-related and non UV-related DNA damages in melanoma cells

Even if the great majority of BRAF, RAS, and NF1 mutations harbored UV signatures (26, 30) they also showed a high burden of non-UV related mutations (31). This suggests that UV has a role in melanoma pathogenesis, but UV-unrelated mutations can play a role as well. The pathogenic contribution of these UV-independent mutations is still to be clarified. In addition, analysis of whole-genome sequences reveals different carcinogenic processes across the CMM subtypes, some unrelated to sun exposure, and extends potential involvement of the non-coding genome in its pathogenesis (31).

## UVR, melanoma and melanins

The protective role of eumelanin is suggested by the evidence that people with dark skin are less prone to develop CMM. Indeed, eumelanin has UV-filtering properties. However, experimental findings have shown that the relationship between CMM and melanins is more complex. The action spectrum of human CMM is unknown but, in the 90 s, Setlow et al. demonstrated that UVA and UVB wavebands have similar pathogenetic activity for CMM in the xiphophorus fish model. Furthermore, they demonstrated that melanin-photosensitized radical production is the major causative step of CMM of this fish (32). Recent experimental work in transgenic mice, confirmed that UVA dependent eumelanin's pro-oxidative activity has a significant pathogenetic role for CMM (33). In addition, unlike UVB, that initiates CMM in a “pigment-independent” manner through direct DNA damage, UVA was found to require the presence of melanin (33). Furthermore, it is worrying to know that, in a preliminary study, it was found that the majority of UVA- induced CPDs in melanocytes are generated after more than 3 h from exposure (34). These “dark CPDs” arise when UVA-induced reactive oxygen and nitrogen species combine to excite melanin that induces CPDs by energy transfer to DNA, in a radiation-independent manner (34). Studies on albino African people provide more evidence that melanin is important for CMM development. The incidence of CMM in this population is low while they still early produce several non melanoma skin cancers (NMSCs).

The highest risk of CMM belongs to people with a ‘red hair/fair skin' phenotype, who synthesize a great amount of phaeomelanin. People with homozygote and heterozygote red hair MC1R variants have eumelanin/ pheomelanin ratio of 1.46 and 4.44 respectively, while wild types have 5.81. Unlike eumelanin, phaeomelanin has poorer protective activity against UVR and greater oxidative potential, both in the dark and after UV exposure (35). Phaeomelanin synthesis is regulated by the MC1R gene. Its variants could play a role in CMM development, also via non-pigmentary pathways (36), including a defective control of α- melanocortin (α-MSH)-mediated DNA repair (37, 38), repair of oxidative DNA damage (39), Nucleotide excision repair system and PTEN- dependent pro-apoptotic pathway (40).

Beside red hair subjects, specific MC1R variants may be also found in people with dark hair. These people have an increased risk to develop CMM. Finally, MC1R regulates the expression of the transcription factor MITF that, in addition to pigment biosynthesis enzymes, regulates genes that control DNA repair (APEX nuclease1) (41), cell cycle (CDKN2A, CDK2) (42, 43), apoptosis (BCL2) (44), and invasion (DIA1) (45).

## UVR, phototoxic drugs and melanoma

Experimental and clinical findings suggest that drugs (e.g., azathioprine, vemurafenib, fluoroquinolone antibiotics, propionic acid derivative NSAIDs and voriconazole) can favor melanomagenesis following activation by repeated sub-erythemal UVA exposure (46–48). Also, high levels of folic acid are claimed to have a genotoxic potential because of their pro-oxidative activity that can be further enhanced by pre-treatment with methotrexate (49). The relevance of these findings in the general population is however still to be elucidated.

## UVR induced metabolic changes in melanoma

UVA radiation can play a very important role at an early stage of metastasis through mechanisms that are not directly depending on DNA damage. After repetitive exposures to low doses of UVA, glycolysis and lactate production are increased (Warburg effect) (50). This effect persists for at least 5 days after the last UV exposure and is associated with an up-regulation of several matrix metalloproteinases (MMP2, MMP3, MMP9, MMP13, MMP15), with a consequent increment of melanoma invasiveness (51, 52). Warburg effect increases the speed of tumoral cell mitosis too, because part of the glycolysis-derived pyruvate can be used for anabolic pathways (amino acid or fatty acid synthesis) (53).

## Photoimmunology

CMM is a potentially highly immunogenic tumor due to its multiple auto-antigens (54). However, UV exposure produces a partial loss of immuno-surveillance by decreasing the number and functionality of antigen presenting cells (both Langerhans and dendritic cells) (55, 56). This leads to a shift of the immune response from Th1 to Th2 (57) and the impairment of the activation of effector T cells and NK-T cells (58). The activation of antigen specific regulatory T cells leads to an antigen-specific suppressive effect on the anti-tumor immune response and it creates an environment where skin tumors can grow (59, 60). However, some degree of immune-surveillance is still preserved as shown by the evidence that the risk for CMM is much higher for patients who are immune-suppressed by drugs. Therefore, the main difference between UVR-induced immunosuppression and drug-induced immunosuppression is probably to be identified in antigen specificity (54, 60).

A key problem is the identification of the UV dose that can be significantly dangerous in humans (61). In early studies, immunosuppression in mice was reached with chronic exposures at erythemal doses (62). Later on, it was proved that a single high irradiation (above the erythemal dose) was also capable of producing the same effect (61).

Afterwards, it was demonstrated that low UVB doses (lower than the MED) - as well as UVA - could promote immunosuppression both in mice (61) and human beings (23, 63–65). Consequently, it seems that immunosuppression can be obtained even while normally walking outdoors, in daylight, during summer. However, everyone is frequently exposed to a very low dose of UV. Therefore, the most important question becomes: how much these exposures are dangerous for CMM development (61)? Chronic low-dose exposures were not found to represent a risk factor for CMM in melano-competent subjects (2). Even more surprisingly, it was recently found that a history of sunny holidays, before CMM diagnosis, was associated with lower mean Breslow thickness (66) and intermittent or regular sun exposures, after CMM diagnosis, were associated with lower mean relapse rates (66, 67). A possible explanation is that low/ physiologic doses of UVR inhibit the adaptive immune system but induce parts of the innate immune system (68). However, UVR effects on the presence and activity of innate immune system cells, e.g., macrophages, tumor associated macrophages (TAMs), dendritic cells (DCs), mast cells and NK cells remain to be explored (69).

## UVR, vitamin D and melanoma

Vitamin D and its receptor polymorphisms might play an important role as risk factors for CMM (70). It is very well known that UV radiation is essential for Vitamin D synthesis, in particular for the photoconversion of 7-dehydrocholesterol to cholecalciferol in the epidermis. The production of this vitamin represents one of the most important beneficial effects of sunlight exposure.

Vitamin D bond to its receptor (VDR) results in the transcription of different genes that play a role in the inhibition of MAPK signaling, the induction of apoptosis and cell-cycle inhibition. Therefore, vitamin D has anti-proliferative and pro-apoptotic effects in many kinds of cells, including melanoma cells (71). Vitamin D has also several other positive effects against melanoma, e.g., increase of tumor suppressor PTEN, increase of metastasis suppressor NDRTG1, anti-inflammatory and anti-angiogenic effects and inhibition of “*in vivo*” melanoma cell proliferation, migration and metastasis (71). However, studies about Vitamin D immunological activity apparently show contrasting findings (61). It was found that Vitamin D might have both a suppressive (58) and a protective activity (72). In other studies it was reported that it is not necessary to immune-suppress UV irradiated animals with Vitamin D to induce CMM (58, 73). A possible explanation of these contrasting effects could be found in different vitamin D concentrations and/or particular pre-activated pathways (58). For example: topically applied 0.1 μg of 1,25(OH)2VitD3 (diluted in acetone/olive oil, 4:1), which represents 240 pmoles, seems able to induce immunosuppression. On the other hand, Dixon et al. (72) used 159.6 and 44.8 pmoles (diluted in ethanol, propylene glycol, and water to a final solvent ratio of 2: 1: 1, resp.) of the vitamin, in order to obtain significant protection against UV-induced immunosuppression. Even though the concentration of Vitamin D used in these experiences is different, an important question arises: which one best represents the concentration of vitamin D in the skin after UV exposure? A conclusive answer has not been found so far (73–75) but it seems likely that biological Vitamin D increments after UV exposure are not sufficient to justify the suppression of specific immune responses. Findings of recent clinical studies have suggested (but by no means proved) that vitamin D might also have a role in melanomagenesis and tendency to metastatic dissemination (71). Godar et al. have suggested that low cutaneous vitamin D3 levels with high environmental and low ratio of UVB/UVA doses are the two main drivers for CMM development. In fact, both Europeans and Americans, in some age groups, have a significant increase of CMM incidence if this ratio decreases (76). If this is true, we could explain the curious relationship between melanoma risk and sun exposure, where sunburn is a factor but occupational sun exposure is not (at least in temperate climes). In MM patients, decreased 25(OH)D serum levels are associated with increased tumor thickness and advanced tumor stage (77).

## Conclusions

**Figure 4:**
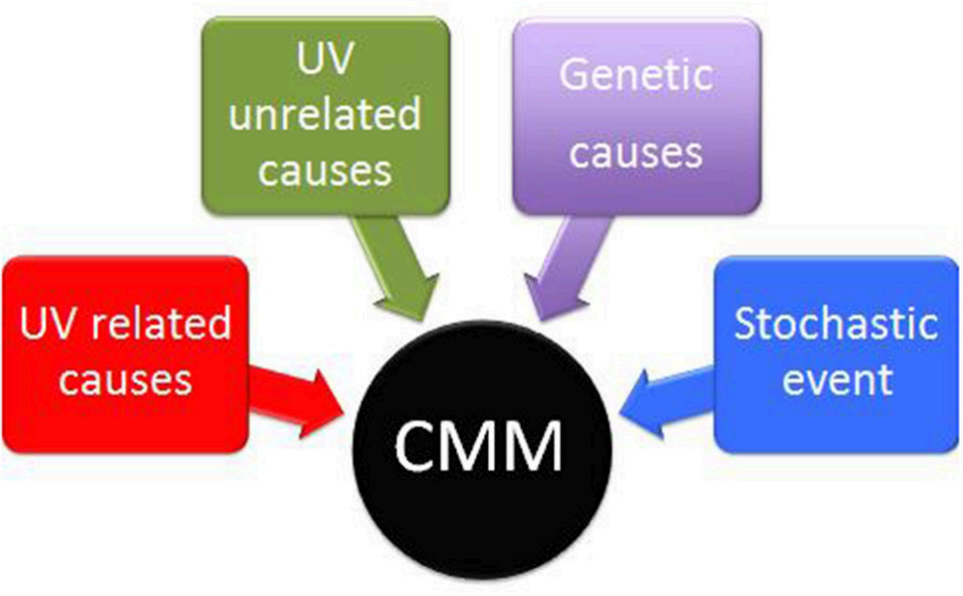
Interplay of UV related, UV unrelated, genetic and stochastic factors in the pathogenesis of cutaneous Malignant Melanoma (CMM).

The life of human beings depends on the sun. This relationship may be beneficial but, at the same time, dangerous. CMM is one of the most deadly tumor and sunlight is, for sure, the main risk factor, although genetic, UV-unrelated and stochastic factors could also play a pathogenetic role (78) (Figure 4). Worldwide, CMM incidence is progressively increasing and two over simplified hypotheses are often put forward as possible explanations: (1) increasing sun exposures and (2) increasing aging of the population. However, we have no data to support the first hypothesis (when exposing to the sun, people tend to be more careful now than before), and the increase of life expectancy in western countries in the last 2 decades seems rather stable^1^. In addition, a growing number of data point out that the relationship between sun exposure and CMM is not simple and straightforward. In particular, even if there is not a light dose that can always be considered either dangerous or beneficial, we know that some UVR doses and some UVA/UVB combinations have a better ratio of beneficial rather than dangerous effects. It is reasonable to conclude that the assessment of the optimal UVR exposure level for each individual will be one of the major future challenges.

## Author contributions

PC-P, MV, MA, AZ, SC, and CZ researched literature to gather necessary information and contributed in the writing of the paper. PC-P, MV, MA, AZ, SC, CR, and CZ reviewed the article several times to reorganize the concepts and provide a more solid structure and reviewed the bibliography.

### Conflict of interest statement

The authors declare that the research was conducted in the absence of any commercial or financial relationships that could be construed as a potential conflict of interest.

## Footnotes

^1^http://www.worldlifeexpectancy.com/world-life-expectancy-map.
